# Discussion of Weight Loss Surgery in Instagram Posts: Successive Sampling Study

**DOI:** 10.2196/29390

**Published:** 2021-11-01

**Authors:** Zoe C Meleo-Erwin, Corey H Basch, Joseph Fera, Bonnie Smith

**Affiliations:** 1 Department of Public Health William Paterson University of New Jersey Wayne, NJ United States; 2 Department of Mathematics Lehman College City University of New York Bronx, NY United States

**Keywords:** bariatric surgery, social media, Instagram, health promotion, post-operative medicine, Instagram, online health information, information accuracy, surgery, information quality

## Abstract

**Background:**

The majority of American adults search for health and illness information on the internet. However, the quality and accuracy of this information are notoriously variable. With the advent of social media, US individuals have increasingly shared their own health and illness experiences, including those related to bariatric surgery, on social media platforms. Previous research has found that peer-to-peer requesting and giving of advice related to bariatric surgery on social media is common, that such advice is often presented in stark terms, and that the advice may not reflect patient standards of care. These previous investigations have helped to map bariatric surgery content on Facebook and YouTube.

**Objective:**

This objective of this study was to document and compare weight loss surgery (WLS)–related content on Instagram in the months leading up to the COVID-19 pandemic and 1 year later.

**Methods:**

We analyzed a total of 300 Instagram posts (50 posts per week for 3 consecutive weeks in late February and early March in both 2020 and 2021) uploaded using the hashtag #wls. Descriptive statistics were reported, and independent 1-tailed chi-square tests were used to determine if a post’s publication year statistically affected its inclusion of a particular type of content.

**Results:**

Overall, advice giving and personal responsibility for outcomes were emphasized by WLS posters on Instagram. However, social support was less emphasized. The safety, challenges, and risks associated with WLS were rarely discussed. The majority of posts did not contain references to facts from reputable medical sources. Posts published in 2021 were more likely to mention stress/hardships of living with WLS (45/150, 30%, vs 29/150, 19.3%; P=.03); however, those published in 2020 more often identified the importance of ongoing support for WLS success (35/150, 23.3%, vs 16/150, 10.7%; P=.004).

**Conclusions:**

Given that bariatric patients have low rates of postoperative follow-up, yet post-operative care and yet support are associated with improved health and weight loss outcomes, and given that health content on the web is of mixed accuracy, bariatric professionals may wish to consider including an online support forum moderated by a professional as a routine part of postoperative care. Doing so may not only improve follow-up rates but may offer providers the opportunity to counter inaccuracies encountered on social media.

## Introduction

It is now well understood that health and illness are key drivers of internet use. Pew Research Center investigators have found that nearly three-quarters of American adults go on the internet to look for information related to health and health care [1]. Over one-third of these individuals use the internet in an attempt to self-diagnose [2]. Notably, high rates of web-based health seeking behavior have been found in other economically advanced countries, such as France [3], Germany [4], and Scotland [5]. In the United States, women, White adults, young people, and those of higher socioeconomic status are most likely to seek diagnoses on the web [6]. Pew Research Center indicates that the most popular web-based health searches include treatments for specific health concerns and conditions [1]. It is therefore unsurprising that the internet has become a primary source of information for individuals who desire weight loss in general [7] and by means of bariatric surgery procedure in particular [8,9]. Furthermore, it seems that as weight increases, so does the likelihood of searching on the web for weight loss solutions [10]. Finally, there is some evidence that individuals interested in bariatric surgery use the internet for health information-seeking more than individuals seeking other forms of digestive system surgery [8].

The (relative) anonymity of internet use may help to explain why those medically diagnosed as overweight or obese—defined by the US Centers for Disease Control [11] as having a BMI of “25.0 to <30” or “30.0 or higher,” respectively—are particularly inclined to use the internet for these purposes. Individuals with stigmatized health conditions have been found to be strong users of the internet for health and illness information searches [12]. Weight-related bias, stigma, and discrimination are considered pervasive in such realms as media, education, employment, and health care [13,14]. This inequitable treatment adversely impacts both the life chances and health outcomes of higher-weight individuals [13-16]. The World Health Organization [17] notes that there is a lack multinational studies comparing weight bias in different countries. However, similar rates of weight bias have been reported in the United States, Canada, Iceland, and Australia [18]. Studies conducted in both the United States [19] and Germany [20] show a positive association between body weight and weight-related bias. Specifically, as an individual’s weight increases, so does the degree of weight-based bias that they experience.

In health care, individuals medically classified as having obesity often face a reduced quality of care, in part due to negative provider attitudes and in part because many health care facilities are not well suited to treat larger-bodied people [13]. Concerns over potential provider bias may lead to delays in seeking care for higher-weight individuals [21]. Thus, the ability to learn more on the web about weight loss in general and weight loss surgery (WLS) in particular may seem to some to be a safer option. Of concern, however, is that the quality and accuracy of health information on the web varies depending on the source. Numerous researchers [22,23] have demonstrated variability in web-based WLS information, noting that the risks of bariatric procedures are not well discussed and the content often lacks provider input.

Just as the advent of the internet has radically shifted the modes by which individuals seek health and illness information, the rise of social media has transformed the ways in which individuals express their own experiences and connect with others around these topics. For example, Pew Research Center investigators [1] have found that going on the web to read or watch videos about others’ health and illness experiences, finding people with similar health concerns, asking health and illness-related questions, and posting about one’s own health and illness experiences are common drivers of social media use, especially for those with one or more chronic health issues. Although the majority of Americans participate in social media, platform use varies by age; older US adults tend to frequent Facebook and YouTube, while younger individuals prefer sites such as Snapchat and Instagram [24].

Researchers have begun to document WLS-related content on social media sites. For example, some investigations have described the nature of WLS support forums on Facebook. Kombrall et al [25] determined that patient posts most commonly pertained to information seeking, provision of tips and advice, and lending encouragement and support to others. In a separate study, Kombrall et al [26] noted that individuals in Facebook WLS patient support groups most commonly solicited and shared nutrition-related advice. Much of the advice given was presented in stark terms (“eat this,” “don’t eat that”), as opposed to the more nuanced and personalized approach that registered dieticians take when advising patients. The authors of an additional investigation [27] found that although Facebook WLS support group membership includes bariatric providers, these individuals had low levels of participation. Recommendations were instead often provided by seasoned patients who positioned their time out of surgery as grounds for providing advice and information to preoperative and newly postoperative individuals. Bariatric professionals do have a presence on YouTube; however, as with Facebook, content is largely driven by lay individuals, and the most popular videos tend to be of lower educational quality [28].

Although these studies have helped to map the nature of WLS content on social media, there remains a dearth of research on Instagram specifically. Instagram is considered to be one of the most commonly used social media sites in operation, with over 1 billion users worldwide [29]. According to the most recent study on social media usage by the Pew Research Center, Instagram is the third most frequently used social media platform in the United States [30]. Globally, Instagram has been ranked fifth in terms of numbers of active users [31]. Instagram users likely span a variety of demographic categories; however, they tend to be younger individuals [23]. For individuals aged 18 to 29 years, Instagram is the second most frequently used social media app in the United States, after YouTube [30]. Instagram is also the social media platform with the second largest age differential between older and younger American users [30]. One investigation found that Instagram users are also disproportionately female, of lower socioeconomic status, and from urban areas [24].

Beyond the general lack of research on this topic, an examination of WLS-related content on Instagram is warranted for a number of reasons. First, although the average age of US bariatric patients has been documented as approximately mid-40s [32], the incidence of WLS in young people increased during the early 20th century [33,34]. Second, individuals between the ages of 18 and 30 years have been found to rely on the internet, including social media, for overall health information [35]. There is evidence to suggest that this age group is more likely to do so than older age cohorts [36]. Third, social media platforms are now commonly used by individuals to seek and relate information related to WLS [7-9; 25-27]. Fourth, seekers of web-based health information do not simply passively take in such information on WLS but actively make decisions based upon what they read on the web [9,22]. These individuals continue to use the internet for postoperative WLS support [9]. Finally, given the shift away from the routine provision of in-person care and support necessitated by the COVID-19 pandemic, bariatric preoperative and postoperative patients may have increasingly turned to social media to fill this gap. This study therefore aimed to document and compare WLS-related content on Instagram in the weeks leading up to the COVID-19 pandemic lockdowns to those exactly 1 year later.

## Methods

The methods used in this study were derived from prior studies that investigated health content posted on social media [37,38]. The sample was comprised of 300 Instagram posts created by users with the hashtag #wls. This hashtag was chosen because it was included in by far the most posts compared to any hashtag associated with weight loss surgery or bariatric surgery (approximately 1 million more posts). The investigation proceeded by means of a successive sampling study: half of the sample was collected in 2020, and the other half was collected exactly 1 year later in 2021. During both 2020 and 2021, 50 posts per week were collected over the course of 3 weeks (the last week of February, the first week of March, and the second week of March), thus minimizing the chance that multiple posts were originating from the same user. Captions, hashtags, and comments made by the user with the same username associated with the post were included in the coding. Posts with photos and videos were included in this study, and any included text was factored into the coding of content. Exclusion criteria included posts that were in a language other than English, images posted without an explanation, and any posts aimed at advertising or selling a product.

A description and the date posted were noted for each post. Each of the posts was studied to see whether it exhibited a predetermined content category. Content coding categories were based on findings of prior research on internet forum and social media use by bariatric surgery patients [25,26,39]. For the data collected, descriptive statistics were recorded, and independent 1-tailed chi squared tests (α=.05) were performed to determine if the year of the post statistically impacted the presence of a given characteristic in the post. Data entry, organization, and analysis were performed in Excel (Microsoft Corporation). The Institutional Review Board at William Paterson University does not require review for studies that involve publicly available social media content. Nevertheless, users may have an expectation of privacy when posting on Instagram; thus, no usernames have been reported in this study.

## Results

Table 1 includes a list of 15 different content characteristics of the studied posts and indicates how many of the 300 posts included this content. The table also includes a breakdown of these counts by post year, with relative percentages indicated.

**Table 1:** Observed characteristics and content of 300 Instagram posts on WLS (150 from 2020 and 150 from 2021).

	Total (N=300), n (%)	2020 (n=150), n (%)	2021 (n=150), n (%)	*P* value
Personal story	275 (91.7)	140 (93.3)	135 (90)	.30
Photo of person	272 (90.7)	127 (84.7)	145 (96.7)	<.001^a^
Identifies type of WLS^b^	120 (40)	95 (63.3)	25 (16.7)	<.001^a^
Gives advice, tips, suggestions	115 (38.3)	63 (42)	52 (34.7)	.19
Stresses personal responsibility for improving health	95 (31.7)	66 (44)	29 (19.3)	<.001^a^
Mentions stress/hardships of living with WLS	74 (24.7)	29 (19.3)	45 (30)	.03^c^
Identifies number of years out of surgery	69 (23)	36 (24)	33 (22)	.26
Identifies importance of ongoing support for WLS success	51 (17)	35 (23.3)	16 (10.7)	.004^d^
Identifies reason for WLS	32 (10.7)	13 (8.7)	19 (12.7)	.26
Identifies ongoing side effects of surgery	25 (8.3)	17 (11.3)	8 (5.3)	.06
Identifies postoperative complications from surgery	23 (7.7)	17 (11.3)	6 (4)	.02^c^
Identifies ongoing work of WLS/WLS as “just a tool”	19 (6.3)	15 (10)	4 (2.7)	.009^d^
States facts	7 (2.3)	1 (0.7)	6 (4)	.06
Discusses weight regain	4 (1.3)	1 (0.7)	3 (2)	.31
Addresses anti-WLS stigma	3 (1)	2 (1.3)	1 (0.7)	.56

^a^Significant at *P*<.001.

^b^WLS: weight loss surgery.

^c^Significant at *P*<.05.

^d^Significant at *P*<.01.

The majority of the posts included a personal story (275/300, 91.7%) and/or included a photo of a person (272/300, 90.7%). Exactly 40% of the posts (120/300) identified the type of WLS, while just under 40% (115/300, 38.3%) gave advice, tips, or suggestions. Just under one-third of the posts (95/300, 31.7%) stressed personal responsibility for improving health. The remaining characteristics were present in less than one-quarter of the posts sampled. The characteristics “gives disclaimer” and “identifies WLS as safe” were not observed in any of the 300 posts; therefore, they are not included in the table.

Independent 1-tailed chi-square tests (α=.05) were performed to determine if a post’s publication year (2020 vs 2021) statistically affected its inclusion of a particular content characteristic. The last column of Table 1 provides the resulting *P* values from these tests, with footnotes indicating statistically significant results with *P*<.05, *P*<.01, or *P*<.001. Compared to 2021, posts made in 2020 more frequently stressed personal responsibility for improving health (55/150, 44%, vs 29/150, 19.3%; *P*<.001), indicated the type of WLS (95/150, 63.3%, vs 25/150, 16.7%; *P*<.001), identified the importance of ongoing support for WLS success (23.3% vs 10.7%; *P*=.004), identified postoperative complications from surgery (17/150, 11.3%, vs 6/150, 4%; *P*=.02), and identified ongoing work of WLS/WLS as “just a tool” (15/150, 10%, vs 4/150, 2.7%; *P*=.009). Posts published in 2021, however, were more likely to include the following content as compared to posts made in 2020: a photo of a person (145/150, 96.7%, vs 127/150, 84.7%; *P*<.001) and mention of stress/hardships of living with WLS (45/150, 30%, vs 29/150, 19.3%; *P*=.03).

Of the 150 posts from 2020, 13 (8.7%) included a reason for the WLS. Of these 13 posts, 6 (46.2%) stated body image as a reason that the user underwent WLS, and 5 (38.5%) indicated health/pregnancy as a reason. In 2021, 19 of the 150 posts (12.7%) included a reason for the WLS. Of these 19 posts, 10 (52.6%) stated body image was a reason the user underwent WLS, and just over 25% of the posts (n=5, 26.3%) indicated health/pregnancy as the reason for undergoing WLS.

## Discussion

This descriptive, successive sampling study documented and compared Instagram content on bariatric surgery in the weeks leading up to the COVID-19 pandemic lockdown to those posted during these same weeks 1 year later. In both years, the majority of individuals who had undergone bariatric surgery did not note their reason for doing so. However, when those reasons were mentioned, they more commonly pertained to body image issues and concerns over future health risks than to current weight-related health problems. Posts containing facts—wherein a user referred to an external medical source of the information contained in the post—were very rare. By contrast, posts offering personal advice, tips, and suggestions for others were far more common. This is understandable, however, given that individuals may use social media to specifically read about the personal experiences of others regarding weight loss surgery and to share their own. Nevertheless, given the variable quality of WLS information on the web [22,23], and given that previous research [25] has found that giving of WLS advice on social media forums is often presented in a more stark and less nuanced manner than a bariatric professional might otherwise provide, it is possible that the uptake of personal advice offered on social media will have negative consequences for bariatric patients. This may be especially the case for younger individuals who have lower levels of health literacy [40,41].

Interestingly, although references to the risks and challenges (eg, complications, side effects, weight regain) associated with WLS were infrequent, discussion of the safety profile of WLS procedures did not occur at all. Personal responsibility for health and weight loss outcomes was stressed more commonly than the importance of social support. Finally, despite research documenting the extensiveness of weight bias, stigma, and discrimination [13,20], as well as research which has found that such stigma extends to an individual’s decision to have bariatric surgery (with WLS patients being accused of having “taken the easy way out” by choosing a surgical means of weight loss) [42,43], psychosocial factors related to either the decision to have WLS or living with such a procedure were uncommon.

Comparing the two years, posters in 2021 were more likely to emphasize the ongoing challenges of living with a WLS procedure but less likely to emphasize personal responsibility for health or the ongoing work required to live with a WLS procedure (to avoid complications, side effects, or weight regain). Moreover, in 2021, posts highlighting the importance of support for postoperative success decreased compared to the year prior. Taken together, it appears that in 2021, bariatric patients posted more often about challenges they faced regarding WLS but less often about intrapersonal or clinical resources that might help address these challenges. This finding is notable given the timing of the data collection. As noted, the first set of data were collected in the weeks immediately preceding COVID-19–related lockdowns in the United States and worldwide. By the time the data were collected 1 year later in 2021, over 500,000 Americans had died of the disease [44]. Globally, there were nearly 2.5 million cumulative deaths from COVID-19 [45]. Additionally, during this time, highly contagious COVID-19 variants were circulating; however, mass vaccination efforts were beginning to move forward. Some preliminary evidence has found that bariatric patient follow-up rates may have been improved by a shift to telemedicine during the pandemic [46]. However, other studies suggest that the pandemic may have increased the risk of adverse physical and mental health outcomes for bariatric patients [47-50]. Clearly, more research will be needed to determine the long-term impact of the pandemic on bariatric patient outcomes.

Arguably, the impact of using the internet to seek health and illness information and connect with others who share concerns and experiences about health and illness is mixed, both in general and regarding WLS specifically. The internet has democratized access to information regarding health and illness (as well as other topics). This democratization has likely enhanced health-related empowerment and self-efficacy and has facilitated patient advocacy, particularly around contested health issues [51]. Additionally, social media forums offer individuals a convenient way to give and receive ongoing support. Given that logistical barriers (eg, availability, time, location, competing responsibilities, and associated expenses) may prevent some individuals from accessing in-person support groups, online support forums can help meet this vital need. In this vein, and given that ongoing support has been found to be associated with improved health outcomes postoperatively [52], we view the use of Instagram and other social media by individuals who undergo WLS to have potential positive benefits.

However, as noted, health and illness information on the web, including that related to bariatric procedures specifically, is of varied quality and accuracy. There does seem to be some debate in the literature regarding whether individuals searching for such information find health information on the web to be credible compared to that from professional sources. What is well known, however, is that the majority of individuals who undergo WLS in the United States do not return for follow-up care and support services within the first 1 to 2 years after surgery [52,53]. Patients may therefore rely on peer-generated information and advice without verifying the accuracy of that information or discussing the appropriateness of applying it to themselves with their bariatric providers.

It is in this the context that we situate the findings of this study and our concern therein that Instagram posters emphasized personal responsibility but not ongoing support for health and weight loss outcomes. More particularly, we are concerned that patients may experience challenges as well as more serious side effects and complications of undergoing WLS but may rely on the application of peer-to-peer advice and information that does not meet standards of care to address these concerns. This may be especially the case for young adults, who have been shown to adopt health and eating behaviors based on Instagram content even when they understand that the posted images and content on the site are highly curated [41]. For our purposes, such curated images may include representations of visual transformation posted without any accompanying fact-based discussion. Young people may also be particularly vulnerable to health advice coming from “influencers” or aspiring influencers whose key purpose for posting social media content is to gain followers and paid sponsorships [54]. Although our study did not assess whether the posters were influencers, given that all posts included in this study were publicly available, it is possible that some of the individuals were posting in this vein.

A number of limitations of this study should be noted. First, in our investigation, we collected data at two moments in time: late February to mid-March 2020, and exactly 1 year later in 2021. Given the constantly changing nature of social media, it is possible that the content would be different at another point in time. Second, this study relied on publicly available posts. It could therefore not capture the nature of posts made by private accounts. However, as noted, our study may have been more likely to capture posts made by influencers and aspiring influencers. Third, we investigated the content of WLS Instagram posts but not the comments. Therefore, we cannot make claims about the conversations that occur between individuals on Instagram regarding WLS. Fourth, we cannot state what impacts, if any, the posts included in the sample had on Instagram users who engaged with them. Fifth, although Instagram is a leading social media site, our study only investigated WLS content on this one platform rather than across social media sites. Sixth, our cutoff of 50 posts at each data collection point was arbitrary in nature. Had we collected a higher number of posts per week, the results may have been different. Seventh, Instagram users in the United States tend to be young people [30]; we were not able to assess the actual age or other demographic characteristics of the posters (via data scraping), in accordance with Instagram’s policies. Finally, although the data were collected just prior to the COVID-19 lockdown period and again 1 year later, we are not able to demonstrate any causal effect of the pandemic on WLS-related Instagram content. Despite these limitations, this study begins to fill a gap in describing how individuals discuss WLS on Instagram.

In this study, we found that visual transformation, advice giving, and personal responsibility for health and weight loss outcomes are emphasized by WLS posters on Instagram. Conversely, social support is less emphasized. The safety, challenges, and risks associated with WLS are rarely discussed, and the majority of posts do not contain references to factual information taken from credible medical sources. Although the safety profile of WLS procedures has improved over time, complications and side effects still occur with some frequency [55]. Moreover, although side effects and complications of WLS are not reducible to patient behavior, they are unquestionably related. Given that American bariatric patients have low rates of postoperative follow-up [52,53], and yet ongoing post-operative support is linked with improved health outcomes [52], offering weight loss surgery patients a variety of convenient and accessible options for accessing care and support is warranted. In 2011, Kaiser et al [56] argued that offering a mix of formats for WLS clinical support groups—telephone, internet, and in-person—may help address barriers to attending in-person group sessions. Offering patients an active online support forum moderated by a bariatric professional may additionally offer the opportunity to counter misinformation that circulates in peer-led forums and may also provide the sort of balanced and nuanced information that can help patients navigate any ongoing challenges of living with a bariatric surgical procedure.

## Abbreviations

WLS: weight loss surgery
